# Influence of length and flexibility of spacers on the binding affinity of divalent ligands

**DOI:** 10.3762/bjoc.11.90

**Published:** 2015-05-15

**Authors:** Susanne Liese, Roland R Netz

**Affiliations:** 1 Fachbereich für Physik, Freie Universität Berlin, 14195 Berlin, Germany

**Keywords:** binding affinity, divalent ligand, effective concentration, multivalency

## Abstract

We present a quantitative model for the binding of divalent ligand–receptor systems. We study the influence of length and flexibility of the spacers on the overall binding affinity and derive general rules for the optimal ligand design. To this end, we first compare different polymeric models and determine the probability to simultaneously bind to two neighboring receptor binding pockets. In a second step the binding affinity of divalent ligands in terms of the IC_50_ value is derived. We find that a divalent ligand has the potential to bind more efficiently than its monovalent counterpart only, if the monovalent dissociation constant is lower than a critical value. This critical monovalent dissociation constant depends on the ligand-spacer length and flexibility as well as on the size of the receptor. Regarding the optimal ligand-spacer length and flexibility, we find that the average spacer length should be equal or slightly smaller than the distance between the receptor binding pockets and that the end-to-end spacer length fluctuations should be in the same range as the size of a receptor binding pocket.

## Introduction

Multivalency is a common design principle in biological systems. The simultaneous binding of several, relatively weakly binding partners is a widely used strategy to strengthen the overall binding affinity [1–3]. Multivalency is believed to play an important role in evolutionary processes, since the collective interaction of several rather simple ligands makes the development of more complex binding partners with a higher binding affinity unnecessary [2]. Also in drug design, the synthesis of artificial multivalent ligands is a promising route to increase the binding affinity or to reduce the amount of substance required for treatment [4–7].

The term multivalency is used for systems that consist of several identical binding partners. Thereby, the larger binding partner, for example a protein, is commonly denoted as receptor, whereas the smaller binding partner, for instance an enzyme or a single molecule, is denoted as ligand. The binding strength of a multivalent structure significantly depends on details of the presentation of ligands and receptors [1]. Each multivalent ligand consists of several monovalent ligands that are connected via a scaffold. The binding affinity of such a multivalent ligand is determined by the interplay between gain in binding energy and loss of entropy associated with conformational degrees of freedom. The more flexible the scaffold is, the better it can adapt to the geometry of the receptor, but the more pronounced on the other hand is the entropy penalty. This simple, qualitative argument shows that the careful choice of the ligand scaffold is essential, in order to benefit from multivalent enhancement. It is therefore desirable to derive a model that allows one to predict the binding affinity of a given ligand-scaffold construct. Several theoretical studies have been dedicated to study the interaction between multi- and polyvalent ligands with receptors arranged on planar surfaces [8–13]. The overwhelming variety of multivalent ligand architectures that range from small divalent ligands to densely packed nanoparticles, led to different approaches to describe multivalency, depending on the size and valency of the system. Several studies aimed to treat ligand–receptor systems with different structures and valencies in the framework of a generalized theory [14–15].

The smallest multivalent system consists of a divalent ligand that interacts with a divalent receptor. Despite its seeming simplicity, the rational design of divalent ligands is still challenging [16–19]. In this paper we examine a general model for a divalent receptor–ligand system in order to estimate the binding affinity from the dissociation constant of the monovalent ligand and the length and flexibility of the ligand spacer.

**Figure 1:**
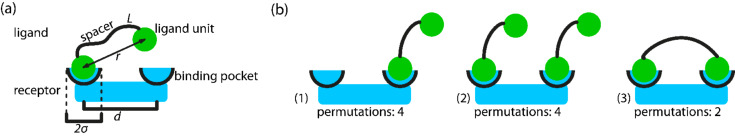
(a) Schematic of a divalent ligand–receptor system: The receptor has two binding pockets with a distance *d* from each other and a binding range σ. The ligand consists of two identical ligand units, connected via a spacer of contour length *L*. The end-to-end distance of the ligand is denoted as *r*. (b) Binding modes of a divalent ligand: (1) One ligand occupies one binding pocket. (2) Two ligands occupy two binding pockets. (3) One ligand occupies both binding pockets.

Figure 1a schematically depicts a divalent ligand–receptor system. The receptor possesses two binding pockets at a distance *d* from each other. A binding range of σ characterizes each binding pocket. The divalent ligand consists of two ligand units that are connected via a spacer. The contour length of the spacer is denoted as *L*. There are three different modes in which a divalent ligand can bind to a divalent receptor. Each of these binding modes has a different number of realization possibilities as summarized in Figure 1b: (1) One binding pocket is occupied by one ligand. (2) Two binding pockets are occupied by two ligands. (3) Two binding pockets are occupied by one ligand. The binding affinity in the latter case is strongly influenced by the conformational linker properties, which can be conveniently discussed in terms of the effective concentration. The effective concentration describes the local concentration of one ligand unit close to one binding pocket, if the other ligand unit is assumed to be bound to the other binding pocket. The effective concentration thus corresponds to the probability that the spacer extends to an end-to-end distance that is equal to *d*, if spacer–receptor interactions are neglected [20]. In the first section different models for the effective concentration are discussed, with particular focus on the influence of the spacer stiffness and the binding range σ.

For each binding mode depicted in Figure 1b the following dissociation constants are derived: (1) The dissociation constant is equal to the dissociation constant of the monovalent ligand, *K*_mono_, multiplied by a factor of 1/α, which accounts for the reduced degrees of freedom of the spacer, since it cannot penetrate the receptor. The parameter α can adopt value between 0 and 1. In the limiting case, in which the spacer sterically inhibits the ligand unit from binding to the receptor, α becomes 0. In the hypothetical case, in which the conformational degrees of freedom of the spacer do not reduce at all when binding to a receptor, the parameter α becomes 1. (2) Each ligand contributes with a factor of *K*_mono_/α to the dissociation constant. (3) The dissociation constant consists of the monovalent dissociation constant for each ligand times the probability that the spacer bridges the two binding pockets. A detailed derivation of the dissociation constants is presented in Supporting Information File 1. Furthermore, Figure 1b summarizes the combinatorial factors for each binding mode that count the number of equivalent permutations. We regard the divalent ligands as distinguishable, we note in passing that this could reflect polymeric spacers that exhibit chemical asymmetry. Our final results do not depend on whether we assume indistinguishable ligand units or not.

## Results and Discussion

### Effective concentration – wormlike-chain model

**Figure 2:**
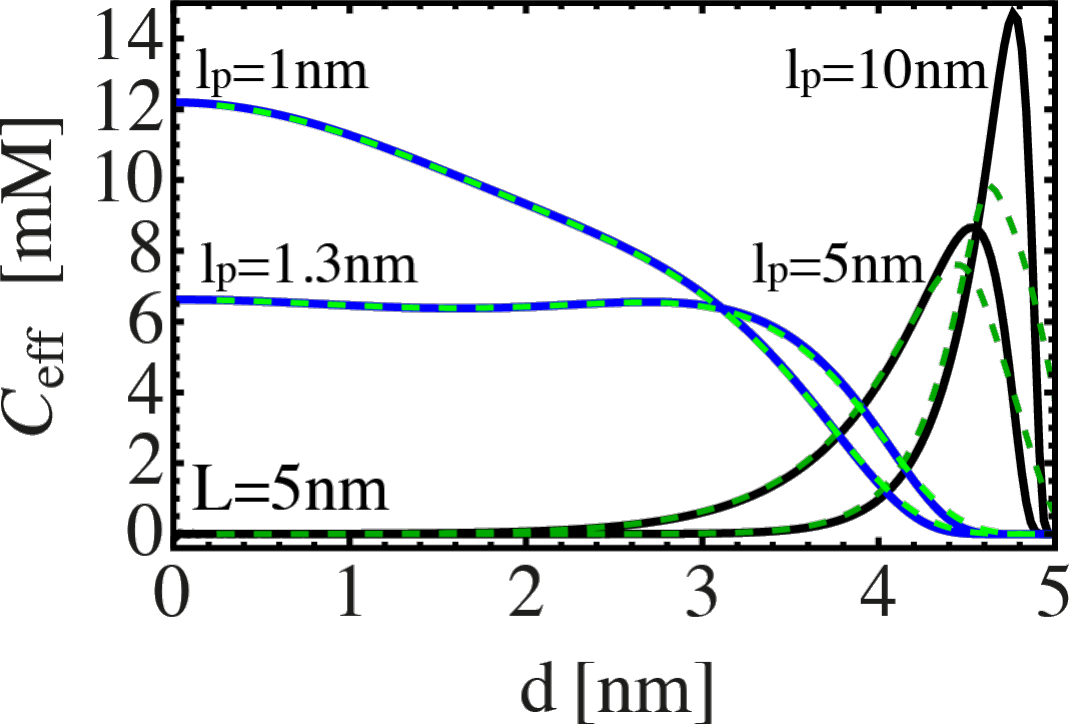
Effective concentration Ceff of spacers with a contour length of *L* = 5 nm as a function of the distance between the binding pockets. The effective concentration is shown for different spacer stiffness, in terms of different persistence lengths between *l*_p_ = 1–10 nm (continuous lines). The effective concentration 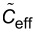, averaged over a binding pocket range σ = 0.25 nm, is shown as green, dashed lines.

Samuel and Sinha [21] developed an exact method to describe the conformational statistics of wormlike chains for the whole range from short to long polymers. Their model is applied here to determine the effective concentration *C*_eff_, which is equivalent to the end-to-end distance probability distribution, with the normalization 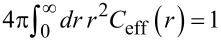. An example is shown in Figure 2. The length of the fully extended spacer *L* is set to 5 nm. The effective concentration, i.e., the probability that a spacer of given length and stiffness extends to a certain end-to-end-distance *d*, is shown for different persistence lengths *l*_p_. The flexible spacer (*l*_p_ = 1 nm) exhibits a maximum at *d* = 0. Furthermore, the distribution is very broad, indicating that a flexible spacer can easily bridge two binding pockets, even if the spacer length does not exactly match the inter binding pocket distance *d*. For a slightly stiffer spacer (*l*_p_ = 1.3 nm), *C*_eff_ is even broader, but the maximum of *C*_eff_ is reduced by a factor of about one half and the distribution shows a plateau between *d* = 0 nm and *d* = 3 nm. For stiff spacers (*l*_p_ = 5 nm and *l*_p_ = 10 nm), *C*_eff_ exhibits a narrow peak close to the fully extended state. In the bound state, the ligand units explore the range σ of a receptor binding pocket. Hence, it is useful to consider the effective concentration averaged over the range of both binding pockets. We denote the averaged effective concentration as 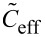 with

[1]
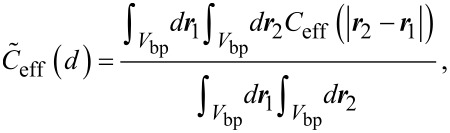


with *V*_bp_ the volume of one binding pocket, **r**_1_ and **r**_2_ the positions within the first and second binding pocket. We introduce the connecting vector **r** = |**r**_1_ − **r**_2_| and express **r** in spherical coordinates:

[2]
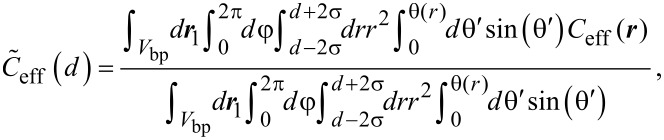


with *r* the distance between the two ligand units, θ the angle between ***r*** and the connecting vector of the binding pocket midpoints and φ an angle that describes the rotation around the connecting vector of the binding pocket midpoints. Since the range of the binding pocket σ is assumed to be much smaller than the distance between the binding pockets *d*, we conclude that the integrals in Equation 2 approximately factorize. Furthermore, the size of the binding pocket limits the range over which the angle θ can vary. In the range, where *r* varies between *d* − σ and *d* + σ, the angle θ can adopt a maximum value of arctan(σ/*r*). The upper limit for the integration over θ then reads


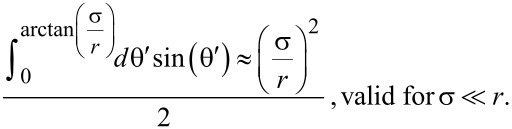


The integration over ***r*** can now be described by variations of *r* in the range from *d* − σ and *d* + σ*.* With these approximations, Equation 2 can be written as an effective average over one dimension:

[3]
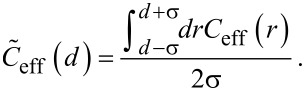


In Figure 2, the averaged effective concentration is shown as green, dashed lines, with σ = 0.25 nm. A flexible spacer can easily extend to all positions within the binding pockets. Hence, one cannot observe any significant difference between 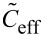 and *C*_eff_. In contrast, a very stiff spacer cannot explore the whole binding pocket. Therefore, the averaged effective concentration is reduced and slightly broadened around its maximum, as can be seen best in Figure 2 for *l*_p_ = 10 nm.

**Figure 3:**
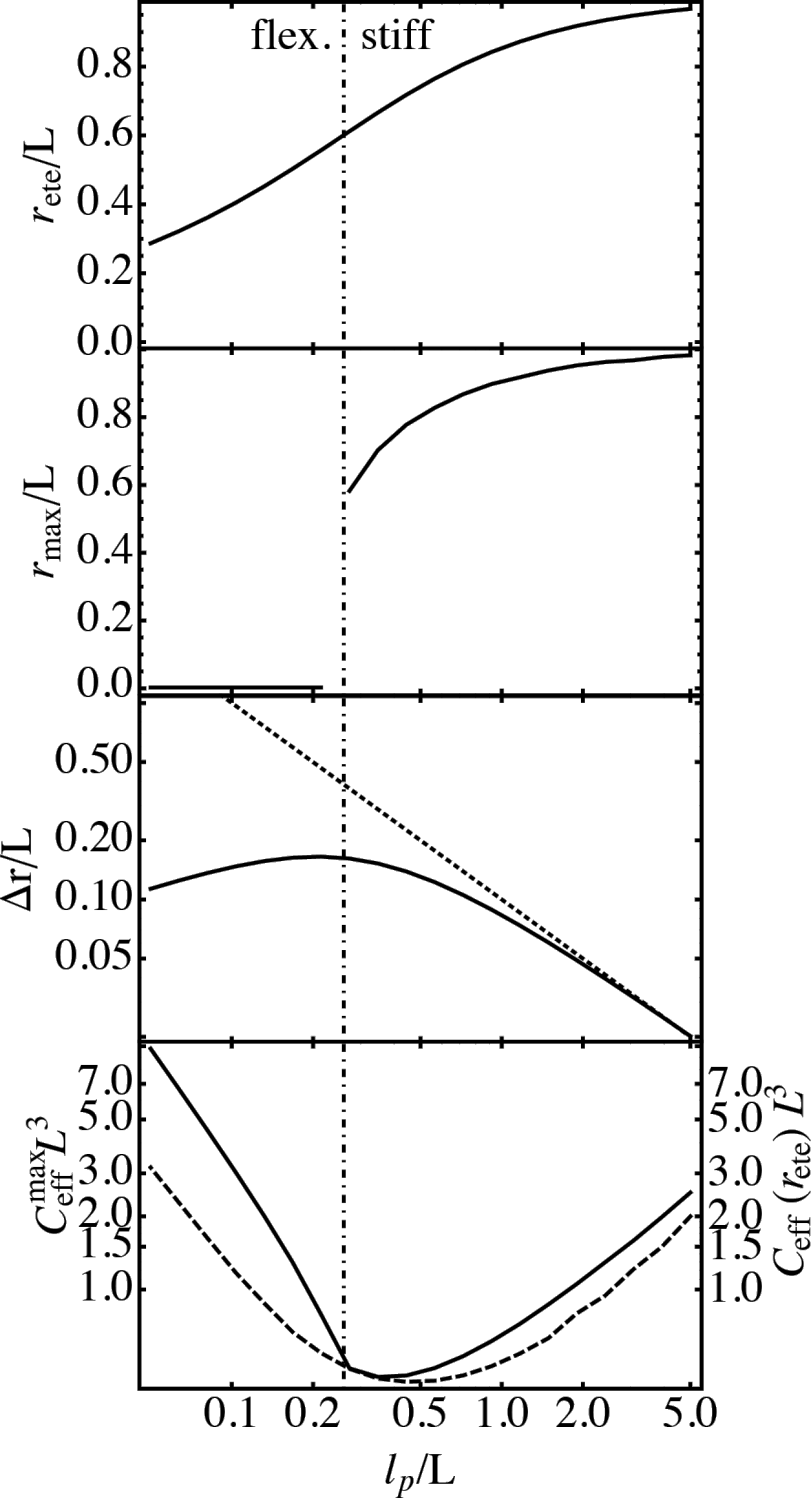
Average end-to-end distance, *r*_ete_, end-to-end-distance where the effective concentration *C*_eff_ exhibits a maximum, *r*_max_, variance of the end-to-end distance distribution, Δ*r*, maximum of the effective concentration, 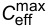 (continuous line), and effective concentration at *r*_ete_, *C*_eff_(*r*_ete_) (dashed line), in dependence of the persistence length *l*_p_. All lengths are measured in units of the spacer contour length *L*. Spacers with a persistence length *l*_p_ < 0.26*L* are called flexible. Spacers with a persistence length *l*_p_ > 0.26*L* are called stiff. For stiff spacers the relation between Δ*r*/*L* and the persistence length is well described by Δ*r*/*L* = 0.1*L*/*l*_p_ (dotted line).

Figure 3 summarizes the averaged end-to-end distance *r*_ete_, the end-to-end distance that corresponds to a maximum in *C*_eff_, *r*_max_, the variance of the end-to-end distance distribution Δ*r*, the maximum of the effective concentration 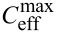 and the effective concentration at *r*_ete_, *C*_eff_(*r*_ete_), for different persistence lengths. The influence of the binding range σ is neglected here. The average end-to-end distance *r*_ete_ increases monotonically with increasing persistence length and approaches the contour length *L* for very stiff spacers. All other quantities reveal a clear-cut difference between the flexible and stiff limits. The classification “flexible” and “stiff” is, of course, to some degree arbitrary. We here apply a definition that is based on the discontinuity in *r*_max_, which is the most prominent feature in the chain observables. In the following, spacers with a persistence length smaller than 0.26*L* are called flexible and spacers with a persistence length larger than 0.26*L* are called stiff. The variance Δ*r* exhibits a maximum around *l*_p_ = 0.26*L*, for stiffer spacers Δ*r* reduces rapidly. As can be seen in Figure 3, the variance Δ*r* depends on the persistence length as Δ*r* = 0.1*L*^2^/*l*_p_ (dotted line) for stiff spacers. Mac Kintosh et al. found the same scaling for the fluctuations of semiflexible polymers [22]. The maximum of the effective concentration 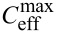 (continuous line) as well as the effective concentration at *r*_ete_, *C*_eff_(*r*_ete_), (dashed line) are minimal in the same region where Δ*r* is maximal. Since for a stiff spacer *r*_max_ and *r*_ete_ are both close to *L*, 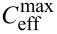 and *C*_eff_(*r*_ete_) exhibit only small deviations from each other. For flexible spacers on the other hand, *C*_eff_(*r*_ete_) can be much smaller than the maximal effective concentration. The results presented here show that neither the persistence length nor the contour length alone are sufficient to describe the behavior of the effective concentration, rather the ratio between persistence length and contour length, *l*_p_/*L*, characterizes the conformational behavior. Note that for a typical receptor distance of *d* = 5 nm, DNA molecules with *l*_p_ = 53 nm are characterized by a ratio *l*_p_/*L* ≈ 10 and thus correspond to the very stiff limit. Polyethylene glycol (PEG) with a persistence length of about *l*_p_ = 0.38 nm on the other hand is characterized by a ratio smaller than *l*_p_/*L* = 0.08 and thus correspond to the flexible limit [23].

### Effective concentration – harmonic spring and Gaussian chain approximation

The wormlike-chain model requires complex numerical analysis for the calculation of conformational chain properties. In a simplified model the spacer statistics can be described as a harmonic spring or a Gaussian chain with suitably chosen parameters. The advantage of this model is that the effective concentration can be derived in closed form. Furthermore, we show that despite its simplified assumptions the model accurately reproduces the effective concentration *C*_eff_(*r*_ete_) for flexible as well as for stiff spacers.

#### Stiff spacer – harmonic spring approximation

A stiff spacer is on average extended to almost its full length. The fluctuations around its most probable end-to-end distance *r*_0_ are assumed to be much smaller than the contour length *L*. We approximate the free energy *F*, similar to a harmonic spring, as

[4]
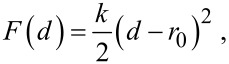


with *k* the effective spring constant and *d* the end-to-end distance. The effective concentration *C*_eff_(*d*), i.e., the normalized probability to extend the spacer to a certain end-to-end distance *d*, reads

[5]
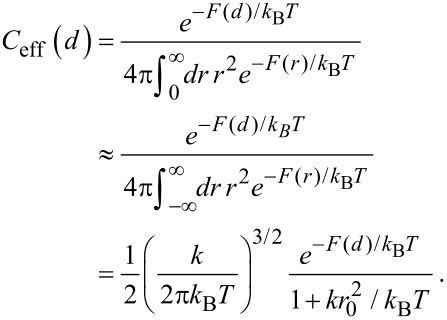


The averaged effective concentration 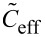 as defined in Equation 3 then becomes:

[6]
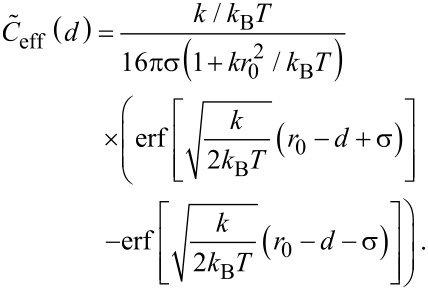


In order to express the effective concentration in term of the experimentally more relevant average end-to-end distance *r*_ete_ and the variance Δ*r*, we first have to determine the relation between *r*_ete_ and Δ*r* on the one side and *k* and *r*_0_ on the other side.

From the free energy *F* in Equation 4 the average end-to-end distance *r*_ete_ and the variance Δ*r* are obtained as:

[7]
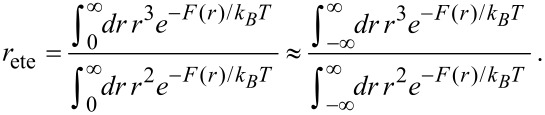


[8]
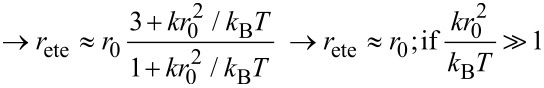


[9]
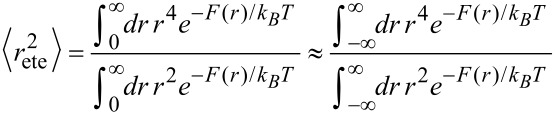


[10]
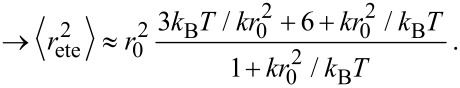


Note that according to our notation, the average end-to-end distance *r*_ete_ is not equivalent to the root mean squared end-to-end distance 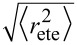. The variance Δ*r* hence reads:

[11]
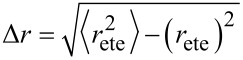


[12]
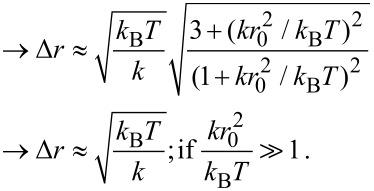


Using Equation 6 and the results for Δ*r* and *r*_ete_ in terms of the model parameters *k* and *r*_0_ in the stiff spacer limit 
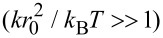
, the averaged effective concentration reads:

[13]
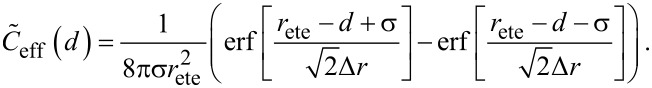


For a fixed distance *d* that has to be spanned by the ligand, the effective concentration becomes maximal for *r*_ete_ = *d* and we obtain, for this optimized spacer length, the result:

[14]
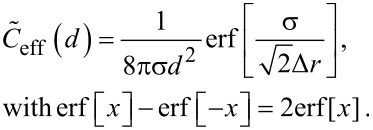


Furthermore, we can differentiate between two cases: 1) the chain fluctuations are smaller than the binding range (Δ*r* << σ) and 2) the chain fluctuations are larger than the binding range (Δ*r* >> σ), leading to

[15]
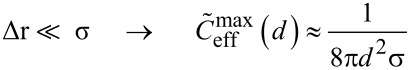


[16]
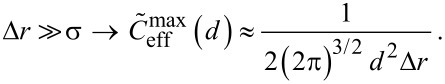


We see that in both limits, the maximal effective concentration decreases quadratically with the distance *d*. More importantly, increasing the stiffness of the spacer (decreasing Δ*r*) increases the effective concentration, but only until the variance Δ*r* becomes of the same order as the binding range σ. For even stiffer spacers the effective concentration stagnates, as can be seen in Equation 15. We conclude that it is not advantageous to increase the spacer stiffness beyond the situation where the end-to-end distance variance Δ*r* becomes smaller than the receptor binding range σ. To compare this model with the wormlike-chain model Equation 16 is rewritten as:

[17]
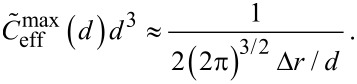


**Figure 4:**
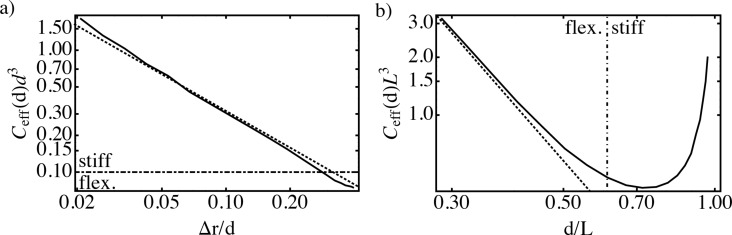
Effective concentration for the optimized average end-to-end distance *r*_ete_=*d* for the wormlike chain model (continuous line) and the harmonic spring model Equation 17 (dotted line, subfigure a) as well as the Gaussian-chain model Equation 18 (dotted line, subfigure b). In the calculation, we vary the ratio between persistence length and contour length *l*_p_/*L*, which results in different ratios Δ*r*/*d* and *d*/*L*, respectively. (a) Stiff spacers are well approximated by Equation 17. (b) Flexible spacers are well approximated by Equation 18.

As can be seen in Figure 4a Equation 17 describes the behavior of stiff wormlike chains very well.

#### Flexible spacer – Gaussian-chain approximation

The effective concentration of flexible polymers is often modeled by a Gaussian chain [11,20,24] with the free energy:

[19]
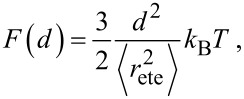


using the mean squared end-to-end distance 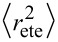. The end-to-end distance *r*_ete_ and the variance Δ*r* can be expressed in terms of the mean squared end-to-end distance:

[20]
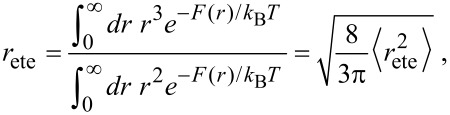


[21]
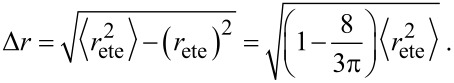


As a consequence the end-to-end distance *r*_ete_ and the variance Δ*r* are related as

[22]
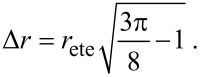


Furthermore, the mean squared end-to-end distance can be written as

[23]
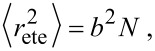


with *b* being the Kuhn length of one chain segment and *N* the number of segments.

We here present the effective concentration as a function of *d* and *r*_ete_.

[24]
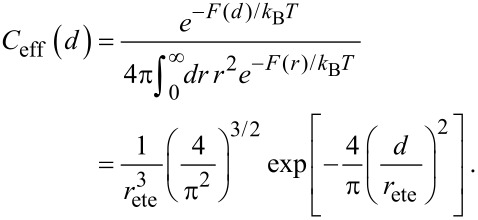


Using Equations 19–22, *r*_ete_ can as well be substituted by 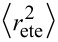, Δ*r* or *N.*

Note that the effective concentration of a flexible spacer with fixed contour length *L* is maximal at a distance *d* = 0, as shown in Figure 2. In contrast, for a given distance *d* the effective concentration becomes maximal at 
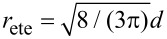
. In other words, the average end-to-end distance of an optimized flexible spacer is smaller than the distance between the binding pockets by a factor of 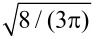:

[25]
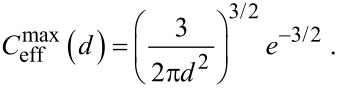


Since we consider the fluctuations of a flexible chain much larger than the range of the binding pocket, we neglect the influence of σ on the effective concentration. In order to compare the behavior of a Gaussian chain with the results for a flexible wormlike chain, Equation 25 is rewritten as:

[18]
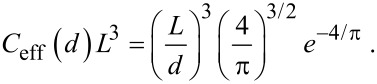


In Figure 4b, Equation 18 is shown together with the numerical results from the wormlike chain model obtained in the previous section. The two models show good agreement in the flexible limit, as expected.

### Conformational degrees of freedom of a tethered spacer

If one ligand unit is bound to one of the binding pockets, the conformational degrees of freedom of the spacer are reduced, since it cannot penetrate the receptor surface. We quantify this reduction by the parameter α, which describes the ratio between the partition function of a tethered and a free spacer. The value of α depends on the shape of the receptor and the flexibility of the spacer. To estimate the typical magnitude of α we consider as limiting cases a stiff rod as well as a flexible Gaussian chain tethered to a planar surface.

#### Stiff spacer

For a stiff rod attached with one end to a planar surface, the parameter α becomes α = 1/2, since the rod can only explore one half space.

#### Flexible spacer

As a second example we discuss a Gaussian chain. Equivalently to Equation 24 the normalized probability that a Gaussian chain consisting of *N* segments extends to an end-to-end distance *r* with *b* being the length of one segment reads in free space:

[26]
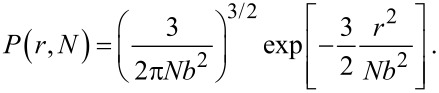


We now assume that one end of the chain is attached to the surface. Similar to the considerations made for a stiff rod, we approximate the probability that the first segment does not penetrate the surface by a factor 1/2. The probability distribution for the remaining *N* − 1 segments then reads:

[27]
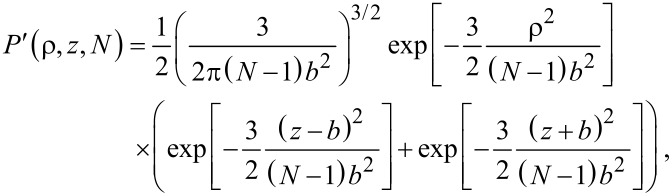


with ρ the component of the end-to-end vector parallel to the surface and *z* the height above the surface. The last term in Equation 27 ensures that the chain does not penetrate the surface (*P*′(ρ,*z* = 0,*N*) = 0). To obtain the parameter α, *P*′ has to be integrated over one half space:

[28]
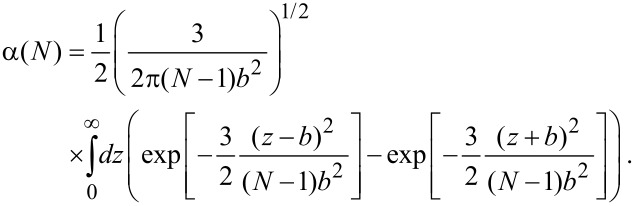


In the limit of a long chain (*N* >> 1), Equation 28 can be approximated as:

[29]
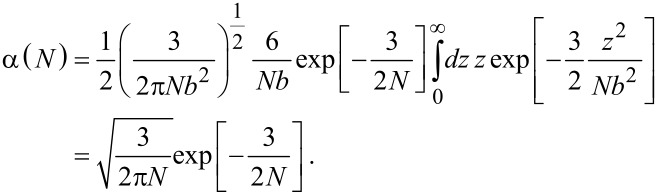


A PEG spacer with *b* = 0.38 nm requires 30–800 segments to adopt an average end-to-end distance of 2 to 10 nm. In this range α varies between 0.02 and 0.13.

### Binding affinity

With the effective concentration and a parameterization for the reduction of the conformational degrees of freedom of the spacer at hand, we now can examine the binding affinity of a divalent ligand. A common way to quantify the binding affinity of a multivalent ligand is the so-called IC_50_ value, the ligand (or inhibitor) concentration at half maximal inhibition. In a first step we want to re-derive the relation between the IC_50_ value and the dissociation constant of a monovalent ligand [25–26].

#### Monovalent ligand

In the reaction 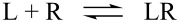, the dissociation constant *K*_mono_ of a monovalent ligand interacting with a monovalent receptor is defined as

[30]
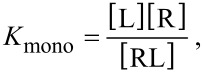


with [L] and [R] being the concentration of unbound ligands and unbound receptors and [RL] the concentration of bound ligands or equivalently the concentration of bound receptors.

If half of all receptors are occupied, which defines the IC_50_ condition, the other half must be unbound and as a consequence [R] = [RL]. From Equation 30 we see that under IC_50_ conditions the dissociation constant equals the concentration of unbound ligands:

[31]
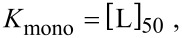


with the index 50 indicating that the IC_50_ condition is fulfilled. In the monovalent case exactly one ligand binds to one receptor. Thus, the concentration of bound ligands under IC_50_ conditions is given by half the total receptor concentration:

[32]
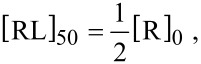


with [R]_0_ = [R] + [RL] the total receptor concentration. Combining Equation 31 and Equation 32 the IC_50_ value is obtained as [25]:

[33]
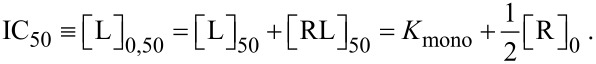


In the limit of dilute receptor conditions ([R]_0_ << *K*_mono_) the IC_50_ value is a good approximation for the dissociation constant, and we find:

[34]
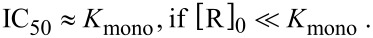


#### Divalent ligand

In analogy to the monovalent case, we now derive an expression for the IC_50_ value of a divalent ligand. There are different ways of defining half maximal inhibition for divalent receptors. We first adopt a heuristic definition where half of all receptor binding pockets are occupied by a ligand unit. This definition is most relevant for competitive binding assays, for instance surface plasmon resonance measurements [27], since the measured signal in a competitive binding assay is related to the number of occupied binding pockets. Later, we also define a situation in which at least one ligand unit is bound to half of all receptors as IC_50_ condition, which mimics non-competitive binding assays, as for instance hemagglutination assays [28]. In non-competitive binding assays the number of bound ligands rather than the number of occupied binding pockets is measured. In general the concentration of occupied binding pockets [bp]_occ_ of divalent receptors reads:

[35]
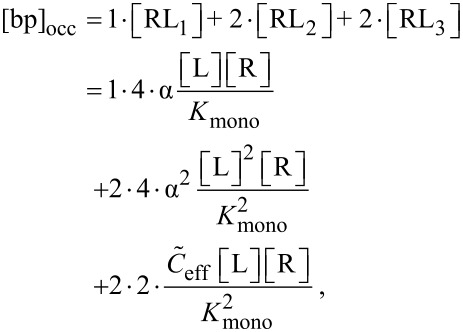


with [RL*_n_*] being the concentration of bound ligand–receptor pairs, with *n* referring to the three binding modes summarized in Figure 1b. Each term on the right hand side of Equation 35 has two prefactors. The first prefactor counts the number of occupied binding pockets per receptor and the second prefactor counts the permutations due to the distinguishability of the ligand units and the receptor binding pockets (see Figure 1b). Note that the number of permutations presented in Figure 1b and Equation 35, are obtained for distinguishable ligand units. For indistinguishable ligand units the number of permutations in each binding mode is reduced. At the same time the dissociation constant of a ligand with indistinguishable ligand units is reduced by the same factor. Hence, the overall concentration of bound ligands does not change. A detailed derivation of the dissociation constants for each binding mode is presented in Supporting Information File 1.

In the same way the total concentration of binding pockets, [bp]_0_, can be obtained as

[36]
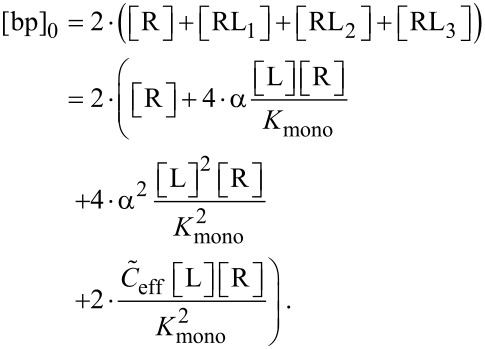


In order to discuss also the IC_50_ condition for non-competitive binding assays we derive the concentration of receptors with at least one binding pocket occupied, [R]_1bp_, and the total receptor concentration, [R]_0,_ as

[37]
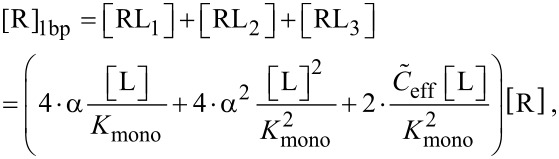


[38]
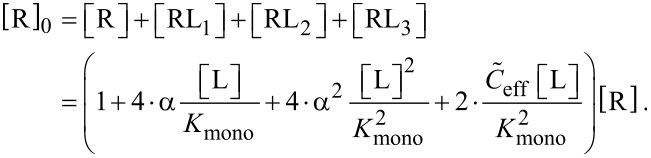


With Equations 35–38 the IC_50_ condition for competitive and non-competitive binding is expressed as given in Equation 39 and Equation 40.

[39]
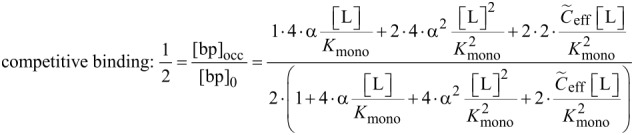


[40]
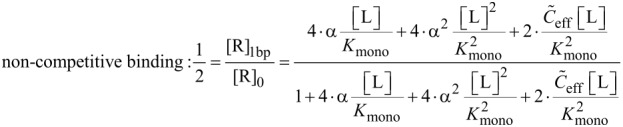


In analogy to the monovalent case we define the multivalent dissociation constant *K*_multi_ as the concentration of free ligand under IC_50_ conditions, as defined in Equation 39 and Equation 40.

Equation 41 and Equation 42 show the multivalent dissociation constant *K*_multi_ in case of competitive binding and non-competitive binding, respectively.

[41]
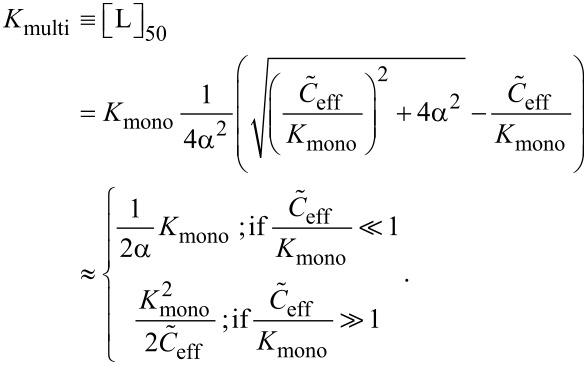


[42]
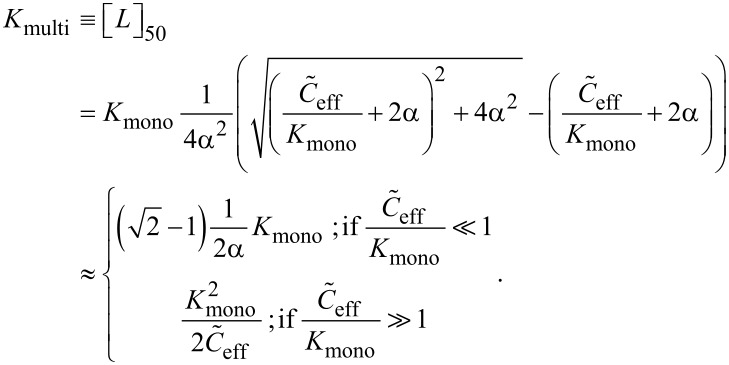


Competitive and non-competitive binding exhibit the same qualitative behavior for large effective concentrations. We therefore limit the further discussion to competitive binding, as given in Equation 41.

As one would intuitively expect, the multivalent dissociation constant *K*_multi_ becomes proportional to the monovalent dissociation constant, if the effective concentration is low, i.e., if 
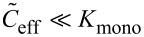
. In contrast, the multivalent dissociation constant decreases, if the dissociation constant of the monovalent ligand is small and if the effective concentration, i.e., the probability to connect two binding pockets, is large.

To determine the total ligand concentration we first have to derive the concentration of bound ligand [L]_bound_ as shown in Equation 43.

[43]
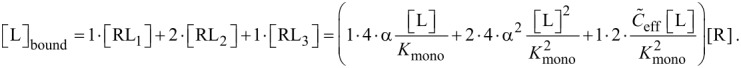


Using Equation 38 and 43, a relation between the concentration of bound ligands and the total receptor concentration under IC_50_ conditions is obtained as

[44]
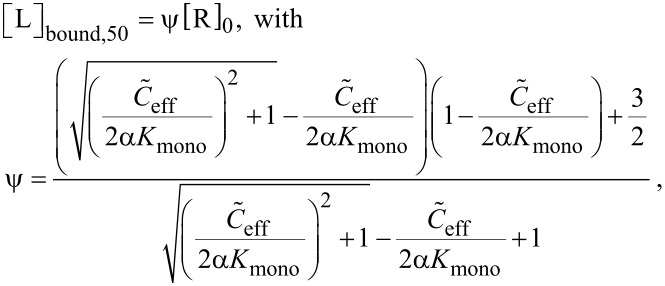


[45]
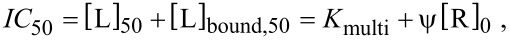


where we note that that ψ is a coefficient that varies between 1 and 5/4. Similar to the results for monovalent receptor–ligand systems in Equation 34, the IC_50_ value becomes equivalent to the multivalent dissociation constant, in the limit of low receptor concentrations, i.e., for [R]_0_ << *K*_multi_:

[46]
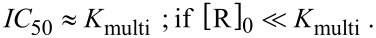


To compare monovalent and multivalent ligands we use the relative binding affinity (*RBA*), which we define as

[47]
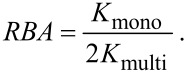


**Figure 5:**
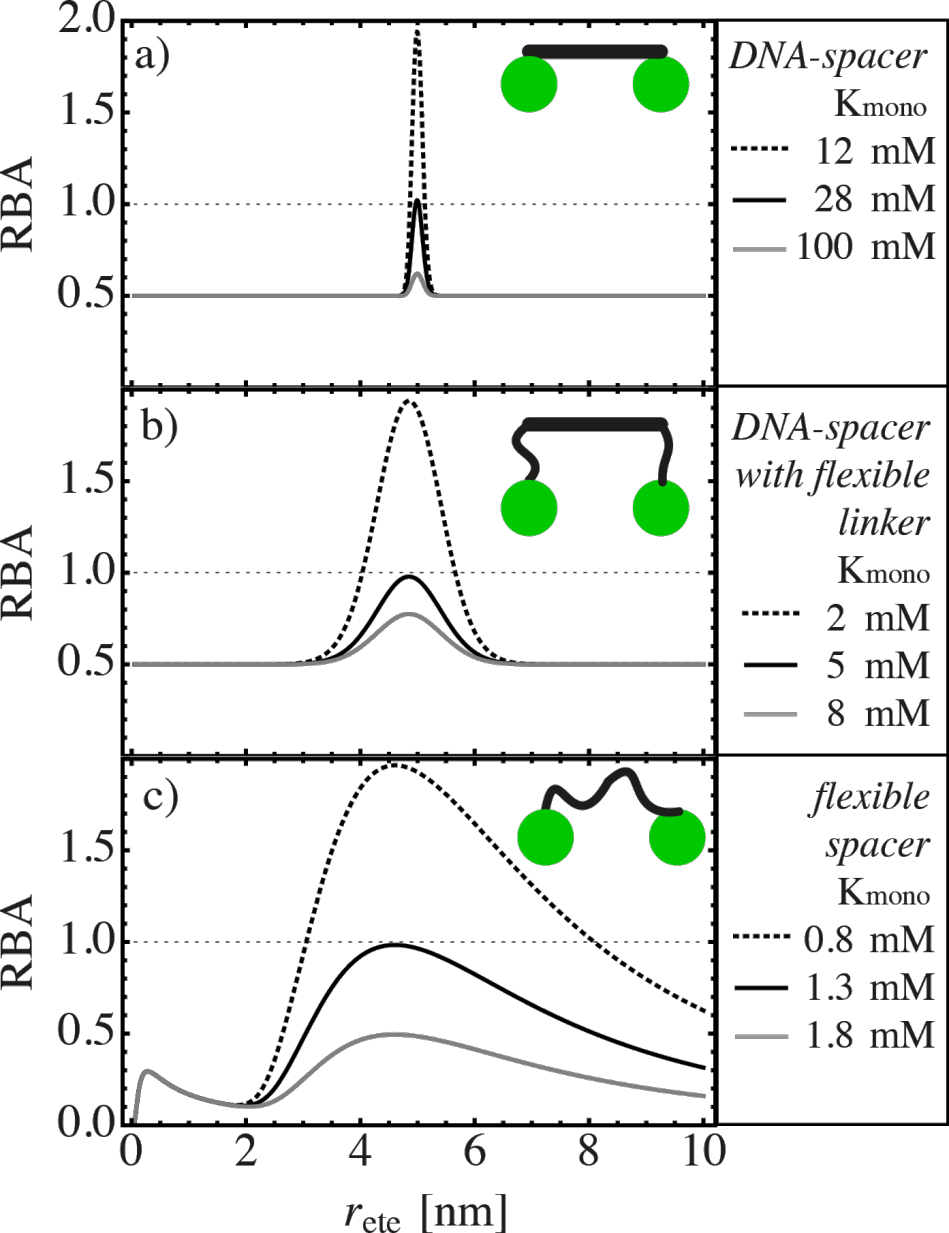
Relative binding affinity (*RBA*) of a divalent ligand in dependence of the end-to-end distance of the spacer *r*_ete_ from Equation 47. The three different ligand–spacer constructs are schematically depicted in the insets. The binding pockets are separated by *d* = 5 nm. Each binding pocket has a binding range of σ = 0.1 nm. (a) The ligand units are directly attached to a stiff DNA spacer, characterized by a persistence length *l*_p_ = 53 nm. (b) The ligand units are attached to a stiff DNA spacer with flexible linker chain, leading to an end-to-end distance fluctuation of Δ*r* = 0.5 nm. (c) The ligand units are connected via a flexible spacer.

Here, the factor 2 accounts for the valency of the ligand and ensures that the concentration of ligand units are compared. The larger the *RBA* the better is the divalent ligand. For *RBA* = 1 the same concentration of mono- and divalent ligand units, taking into account that a divalent ligand consist of two ligand units, is required to occupy half of the receptor binding pockets. For *RBA* < 1 the monovalent ligand binds better than the divalent ligand. In this case the loss in entropy of the spacer is larger than the gain in binding energy due to the multiple binding of ligand units. Inserting the effective concentration from Equation 13 and Equation 24 into Equation 41 and Equation 47, the *RBA* can be calculated for any given divalent ligand–receptor pair. As an example the *RBA* is depicted for different spacers and different values of *K*_mono_ in Figure 5. We here assume that the receptor is well described by a large, planar surface. Hence, the parameter α is approximated by 1/2 for stiff spacer and by Equation 29 for flexible spacers. In all cases we consider a divalent receptor with a distance *d* = 5 nm between the binding pockets. Each binding pocket has a binding range σ = 0.1 nm. In all three subfigures we see that if *K*_mono_ is too large, i.e., if the monovalent binder is too weak, the *RBA*-value never reaches 1. In such a situation, using the *RBA*-value as a quantifier, the monovalent ligand binds always better than the divalent ligand. Furthermore, at a certain *K*_mono_, which we will further on denote as 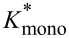, there is exactly one spacer length, parameterized by *r*_ete_, for which monovalent and divalent ligands bind equally well. If *K*_mono_ is lower than 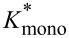, there is a broader range of spacer lengths for which the divalent ligand binds better than the monovalent ligand (*RBA* > 1). In Figure 5a the behavior of a stiff spacer with persistence length *l*_p_ = 53 nm is depicted, which mimics a DNA spacer to which the ligand units are directly attached. A DNA spacer with a contour length of 5 nm exhibits fluctuations in the range Δ*r* ≈ 0.05 nm, which is considerably smaller than the binding range σ. As is discussed in the previous section, the maximum and width of the effective concentration and therefore also the maximum and width of the *RBA* are in this case determined by the binding range σ. In Figure 5b we assume a DNA spacer that is decorated with flexible PEG linkers at both ends. The PEG linkers consist of four monomers each. Assuming Gaussian-chain behavior with a segment length of *b* = 0.38 nm [29], the fluctuations of the PEG linkers and hence the fluctuations of the whole ligand sum up to Δ*r* = 0.5 nm. The shape of the *RBA* now is much broader, showing that the ligand is less affected by a mismatch between spacer length and distance between the binding pockets. Additionally, we obtain 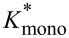 = 5 mM in Figure 5b which is considerably smaller than 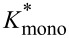 = 28 mM for the pure DNA spacer in Figure 5a. The same trend is continued in Figure 5c. The more flexible the spacer, the smaller is 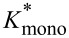, indicating that flexible spacers are less suitable to improve the binding affinity of weak monovalent binders, even though they are more tolerant with respect to a mismatch between linker length and receptor distance.

To investigate the transition from *RBA* < 1 to *RBA* > 1 further, we determine the critical dissociation constant 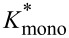 for which the *RBA* is equal to one for the optimized chain length, i.e., for the chain length that maximizes the *RBA* value. Using Equation 41 and Equation 47 it can easily be seen that 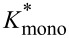 relates to the effective concentration 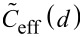 as

[48]
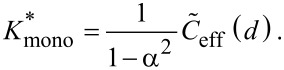


**Figure 6:**
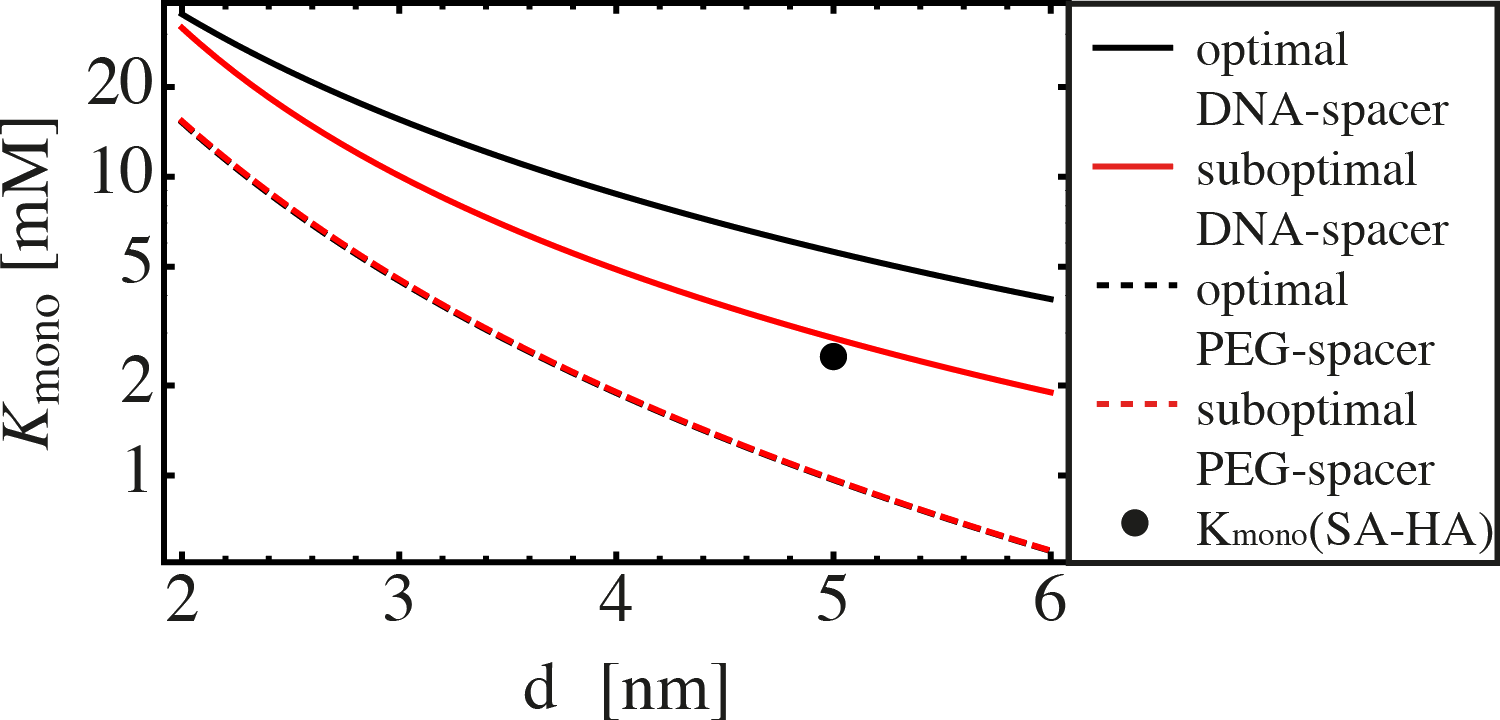
Efficiency diagram: 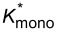 is shown for different ligand–spacer constructs. If the monovalent dissociation constant is larger than 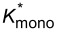, a monovalent ligand always binds better than a divalent ligand. If, on the other hand, the monovalent dissociation constant is smaller than 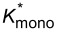, a divalent ligand of suitably chosen length binds better than its monovalent counterpart. We present 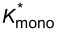 in dependence of the distance between the binding pockets for a DNA spacer with flexible PEG linkers (Δ*r* = 0.5 nm). In the optimal case, the spacer length is chosen equal to the distance *d* (black, continuous line). In the slightly suboptimal case, the spacer length is chosen to be 0.7 nm (two base pairs) shorter than the distance *d* (red, continuous line). In both cases the binding range is set to σ = 0.1 nm. We also show 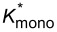 for a flexible PEG spacer with optimized spacer length (black, dashed line) and a spacer that is two monomers shorter (≈0.76 nm) (red, dashed line). The monovalent dissociation constant 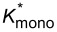 as well as the distance between neighboring binding pockets for a SA–HA pair is indicated by a black point.

In Figure 6, 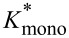 is shown for stiff as well as flexible ligands. The stiff ligand is considered to consist of a DNA spacer to which the ligand units are attached via two PEG linkers. Linker length and binding range are set to be identical to the example presented in Figure 5b. The average end-to-end distance of the DNA spacer is either chosen to be equal to *d* (black, continuous line), or is chosen to be too short by 0.7 nm, which mimics the length of two base pairs (red, continuous line). Even though the mismatch between spacer length and binding pocket distance is small, the ligand becomes significantly less efficient.

The flexible ligand is chosen to resemble a PEG spacer. Again, we assume Gaussian-chain behavior with a segment length of *b* = 0.38 nm. A ligand with optimized spacer length (black, dashed line) does not exhibit a significant difference to a ligand with a spacer that is shortened by two segments (red, dashed line). This shows again that a flexible chain is more tolerant with respect to a distance mismatch between inter-binding pocket distance *d* and chain length.

If the monovalent dissociation constant is larger than 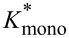, a monovalent ligand always binds better than a divalent ligand. On the other hand, if the monovalent dissociation constant is smaller than 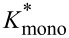, a divalent ligand of optimally (or slightly suboptimal) chosen size binds better than a monovalent ligand.

As can be seen in Figure 6, 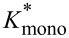 depends on the distance between the binding pockets as well as the spacer length and flexibility. In order to approximate an upper limit for 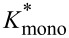, the maximum effective concentration (Equation 25 for a flexible spacer and Equation 15 and Equation 16 for a stiff spacer) is substituted into Equation 48:

[49]
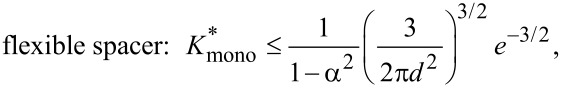


[50]
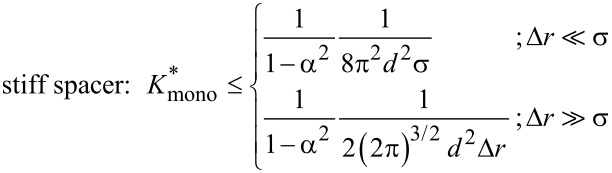


As an example that is relevant for medical applications we want to briefly discuss the interaction between hemagglutinin (HA), a receptor protein on the surface of influenza viruses, and its ligand sialic acid (SA). The dissociation constant between monomeric SA and trimeric HA is known to be 2.5 mM [1]. Furthermore, the crystal structure of HA [30] indicates a distance between neighboring binding pockets in the range of *d* = 5 nm. Note that HA is a trivalent receptor, which means that additional binding modes as well as different numbers of permutations (see Figure 1b) have to be considered. Nevertheless, since the efficiency of a divalent ligand is mainly influenced by the effective concentration 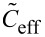 and the monovalent dissociation constant *K*_mono_, rather than by the number of binding modes, we can compare the values for the SA–HA pair with the results presented in Figure 6. We see that a divalent ligand consisting of two SA units connected via a PEG spacer is expected to bind less efficient than the monovalent SA. In contrast, a stiff DNA spacer can increase the binding affinity of the divalent ligand compared to the monovalent ligand, if its length is optimized.

## Conclusion

In the present work we first examine different polymeric models for the effective concentration. We find that a wormlike-chain model can be well reproduced by a simple harmonic spring model and a Gaussian-chain model with suitable chosen parameters, in the stiff and flexible limits, respectively. We next study the binding between divalent ligand–receptor pairs. We find that multivalency increases the overall binding affinity only, if the monovalent ligand–receptor pair binds strongly enough, i.e.; if the monovalent dissociation constant is smaller than a critical value 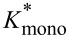. Approximations for 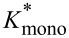 for both flexible and stiff ligands are derived in dependence of the distance between the binding pockets and the spacer length and flexibility. For the optimal ligand design, we find that for stiff ligands the average end-to-end distance should be equal to the distance between the binding pockets and the average fluctuations should be of the order, but not smaller, than the binding range. The average end-to-end distance of a flexible ligand on the other side should be smaller by a factor of 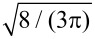 than the binding pocket distance *d*.

## Supporting Information

File 1 Detailed derivation of the dissociation constants for three different binding modes of a divalent ligand.
