# Supplementary material for: Influence of length and flexibility of spacers on the binding affinity of divalent ligands
Source: Beilstein J Org Chem. 2015 May 15;11:804–16. doi: 10.3762/bjoc.11.90 (PMC4464470; doi:10.3762/bjoc.11.90)
Supplement: File 1 — Detailed derivation of the dissociation constants for three different binding modes of a divalent ligand. [file Beilstein_J_Org_Chem-11-804-s001.pdf]

Supporting Information

for

Influence of length and flexibility of spacers on the binding affinity of divalent ligands

Susanne Liese and Roland R. Netz*

Address: Fachbereich für Physik, Freie Universität Berlin, 14195 Berlin, Germany

Email: Roland R. Netz* - rnetz@physik.fu-berlin.de

* Corresponding author

Dissociation constants

The dissociation constant is determined in analogy to the derivations presented by Diestler et al. [1]. We assume that the ligand and receptor concentration is low enough, such that they can be treated as ideal gases. The dissociation constant is then derived as

$$K_d = \frac{(q_R/V)(q_L/V)}{q_{RL}/V} \tag{S1}$$

with q_R, q_L the partition function of the free receptor and the free ligand, q_{RL} the partition function of the bound ligand and V the volume of the system. This notation applies for both monovalent and multivalent systems. In a first step an expression for the monovalent dissociation constant is determined. Afterwards the dissociation constants for different binding modes of a divalent ligand are derived.

Monovalent ligand:

The partition function of the free monovalent ligand is given by the product of the partition function representing the internal degrees of freedom of the unbound ligand $q_{L,i}$ and the volume of the system [2]:

$$q_L = q_{L,i} \int_V \, d\boldsymbol{r} = q_{L,i} V, \tag{S2}$$

with $\boldsymbol{r}$ the position of the monovalent ligand. The partition function of the bound monovalent ligand reads:

$$q_{RL} = q'_R q'_{L,i} \int_{V_{bp}} d\boldsymbol{r} \exp[-F_{\text{bind}}] = q'_R q'_{L,i} V_{bp} \exp[-F_{\text{bind}}], \tag{S3}$$

with q'_R the partition function of the receptor in the bound state and $q'_{L,i}$ the internal partition function of the bound ligand. Here, we approximate the binding energy F_{bind} to be constant throughout the whole volume of the binding pocket V_{bp}. From the partition functions in Equation S2 and Equation S3, the monovalent dissociation constant in Equation S1 is obtained as

$$K_{\text{mono}} = \frac{q_{L,i} q_R}{q'_{L,i} q'_R} \frac{1}{V_{bp}} \exp[F_{\text{bind}}] \tag{S4}$$

Divalent ligand:

The partition function of the free divalent ligand with distinguishable ligand units reads:

$$q_L = q_{L,i}^2 \int_V \, d\boldsymbol{r}_1 \int_V \, d\boldsymbol{r}_2 \exp[-F(\boldsymbol{r}_1 - \boldsymbol{r}_2)] = q_{L,i}^2 V \int_V \, d\boldsymbol{r}_{1,2} \exp\left[-F(\boldsymbol{r}_{1,2})\right], \tag{S5}$$

with $\boldsymbol{r}_1$, $\boldsymbol{r}_2$ the position of the first and second ligand unit, $\boldsymbol{r}_{1,2}=\boldsymbol{r}_1-\boldsymbol{r}_2$ and F the free energy of the spacer. We here assume that the internal partition function of a single ligand unit is equal to the internal partition function of a monovalent ligand $q_{L,i}$. For a ligand with indistinguishable ligand units the partition function q_L reduces by a factor of 1/2. The partition function if one ligand unit is bound (binding mode 1 in Fig1b) in the main text) reads:

$$q_{RL_1} = q'_R q'_{L,i} \int_{V_{bp}} d\boldsymbol{r}_1 \int_{V_{eR}} d\boldsymbol{r}_2 \exp[-F(\boldsymbol{r}_1, \boldsymbol{r}_2) - F_{\text{bind}}] \approx \alpha q'_R q'_{L,i} V_{bp} \exp[-F_{\text{bind}}] \tag{S6}$$

The spacer cannot penetrate the receptor, indicated by the integration over V_{eR}, the volume of the system excluding the receptor, which is modeled by a half space. The reduction of the accessible volume is denoted by the factor α, which can adopt value between 0, if the conformation of the spacer sterically inhibits the ligand unit from binding, and 1, in the hypothetical case that the receptor does not reduce the degrees of freedom of the spacer at all.

From the partition function the dissociation constant in the first binding mode (Figure 1b) in the main text) follows as:

$$K_{d1} = \frac{(q_R/V)(q_L/V)}{(q_{RL_1}/V)} = \frac{[L][R]}{[RL_1]} = \frac{1}{\alpha} K_{\text{mono}}, \tag{S7}$$

with the index 1 refering to the first binding mode.

We furthermore assume that the binding of two ligands occurs independent from each other. Hence, the dissociation constant for two ligands bound to one receptor (second binding mode, Figure 1b) in the main text) reads:

$$K_{d2} = \frac{(q_R/V)(q_L/V)}{(q_{RL_2}/V)} = \frac{[L][R]}{[RL_2]} = \frac{1}{\alpha^2} K_{\text{mono}}^2 \quad \text{(S8)}$$

In the third binding mode (Figure 1b) both ligand units are bound to one receptor. The partition function reads:

$$q_{RL_3} = q_R'' \left(q_{L,i}'\right)^2 \int_{V_{bp}} d\boldsymbol{r}_1 \int_{V_{bp}} d\boldsymbol{r}_2 \exp[-F(\boldsymbol{r}_1 - \boldsymbol{r}_2) - 2F_{\text{bind}}], \quad \text{(S9)}$$

with q''_R the partition function of the receptor, if both binding sites are occupied. Since we assume that the binding processes to two neighboring binding sites are independent from each other, the partition function of the receptor can be written as $q''_R = q_R(q'_R/q_R)^2$. Thereby the factor q'_R/q_R accounts for the change of the internal partion function of each binding pocket. Note that we neglect the influence of the receptor surface on the conformational degrees of freedom of the linker, which is exact in the stiff limit. Equation S9 is now written as

$$q_{RL_3} = q_R \left(\frac{q_R'}{q_R} q_{L,i} V_{bp} \exp[-F_{\text{bind}}]\right)^2 \frac{\int_{V_{bp}} d\boldsymbol{r}_1 \int_{V_{bp}} d\boldsymbol{r}_2 \exp[-F(\boldsymbol{r}_1 - \boldsymbol{r}_2)]}{\int_{V_{bp}} d\boldsymbol{r}_1 \int_{V_{bp}} d\boldsymbol{r}_2} \quad \text{(S10)}$$

From this equation the following dissociation constant is obtained:

$$K_{d3} = \frac{(q_R/V)(q_L/V)}{(q_{RL_3}/V)} = \frac{[L][R]}{[RL_3]} =$$

$$\frac{K_{mono}^2}{\tilde{C}_{\text{eff}} d, \boldsymbol{r}_{1,2}, \sigma)} \quad with \quad \tilde{C}_{\text{eff}}(d, \boldsymbol{r}_{1,2}, \sigma) = \frac{\int_{V_{bp}} d\boldsymbol{r}_1 \int_{V_{bp}} d\boldsymbol{r}_2 \frac{\exp[-F(\boldsymbol{r}_1 - \boldsymbol{r}_2)]}{\int_V d\boldsymbol{r}_{1,2} \exp[-F(\boldsymbol{r}_{1,2})]}}{\int_{V_{bp}} d\boldsymbol{r}_1 \int_{V_{bp}} d\boldsymbol{r}_2}, \quad \text{(S11)}$$

with $\tilde{C}_{\text{eff}}$ the averaged effective concentration concentration, which depends on the length and flexibility of the spacer as well on the distance between the binding pockets *d* and the binding range of each binding pocket σ.

References

1. Diestler, D. J.; Knapp, E. W. *J. Phys. Chem.* **2010,** *114,* 5287–5304.

2. Leunissen M. E.; Dreyfus R.; Sha R.; Seeman N. C.; Chalkin P. M. *J. Am. Chem. Soc.* **2010,** *132,* 1903–1913.
